# Role of P38 MAPK on MMP Activity in Photothrombotic Stroke Mice as Measured using an Ultrafast MMP Activatable Probe

**DOI:** 10.1038/srep16951

**Published:** 2015-11-19

**Authors:** Di Chang, Yuan-Cheng Wang, Ying-Ying Bai, Chun-Qiang Lu, Ting-Ting Xu, Lei Zhu, Shenghong Ju

**Affiliations:** 1 Jiangsu Key Laboratory of Molecular and Functional Imaging, Department of Radiology, Zhongda Hospital, Medical School of Southeast University, Nanjing 210009, China; 2 State Key Laboratory of Molecular Vaccinology and Molecular Diagnostics & Center for Molecular Imaging and Translational Medicine, School of Public Health, Xiamen University, Xiamen, Fujian, 361005, China

## Abstract

Matrix metalloproteinases (MMPs) exert a dual effect in ischemic stroke and thus represent an ideal target for detection and therapy. However, to date, all clinical trials of MMP inhibitors have failed, and alternative drug candidates and therapeutic targets are urgently required. Nonetheless, further investigations are limited by the lack of non-invasive imaging techniques. Here, we report a novel, fast and ultrasensitive MMP activatable optical imaging probe for the dynamic visualization of MMP activity in photothrombotic stroke mice. This probe provides a significant signal enhancement in as little as 15 min, with the highest signal intensity occurring at 1 h post-injection, and shows high sensitivity in measuring MMP activity alterations, which makes it specifically suitable for the real-time visualization of MMP activity and drug discovery in preclinical research. Moreover, using this probe, we successfully demonstrate that the regulation of the p38 mitogen-activated protein kinase (MAPK) signal pathway is capable of modulating MMP activity after stroke, revealing a novel regulatory mechanism of postischemic brain damage and overcoming the limitations of traditional therapeutic strategies associated with MMP inhibitors by using a non-invasive molecular imaging method.

Although stroke is one of the leading causes of death and disability worldwide, very few therapeutic measures and drugs are available to treat this disease^1^,^2^. After ischemic stroke, the increased expression of matrix metalloproteinases (MMPs) contributes to the disruption of the blood-brain barrier (BBB) and promotes brain edema and hemorrhage^3^. Among all of the MMP subtypes, MMP-2 and MMP-9 are the most investigated in ischemic stroke, and MMP-9 is the principal culprit in BBB disruption and neuron damage^4^. Numerous MMP inhibitors have proven to be effective in blocking the breakdown of tight junction proteins in basic research of ischemic stroke^5^,^6^. However, to date, all clinical trials of these drugs have failed. The failures are mainly due to dose-limiting toxicities and poor knowledge about the complexity of MMP functions in multiple biological processes^7^,^8^,^9^. Nonetheless, given their profound effects in inflammatory diseases, MMPs are still considered both detection biomarkers and therapeutic targets in ischemic stroke, though the lack of appropriate detection methods remains a key obstacle limiting further research.

To improve our spatiotemporal understanding of the complex roles of MMPs in stroke, non-invasive visualization and quantification of MMP activity are of great interest in basic research and clinical practice. In recent years, targeted molecular imaging has made great progress in diagnosis and image-guided therapy^10^,^11^. Among all of the imaging modalities, optical imaging shows the most promise due to its high sensitivity, low cost, non-radioactive irradiation and operating convenience. Near-infrared fluorescence (NIRF) imaging, which utilizes a wavelength of 650–900 nm, is attractive because it has minimal autofluorescence and offers both a deep penetrating depth and low phototoxicity, properties that are particularly suitable for *in vivo* studies^12^. A few MMP activatable NIRF probes have been applied to the detection of MMP expression in tumors and stroke. However, these probes either have a long activation time^13^,^14^ or are undetectable using non-invasive methods^15^,^16^ and are thus not suitable for the real-time evaluation of MMP activity after ischemic stroke.

Considering the critical involvement of MMPs in stroke progression and the ineffectiveness of MMP inhibitors in clinical trials, new MMP inhibitors with modified structures and functions are now being developed to alleviate the side effects of current MMP inhibitors. Further, explorations of novel inhibitory molecules that regulate MMP activity will also provide valuable mechanistic insights into the generation of new neuroprotective therapies for ischemic stroke. Recent data indicate that the p38 mitogen-activated protein kinase (MAPK) signal transduction pathway is involved in MMP regulation in cancer metastasis^17^,^18^. Moreover, the use of p38 MAPK inhibitors, which could effectively avoid the side effects of regular MMP inhibitors, has already been tested in clinical trials for safety and efficacy in inflammation and cardiovascular diseases^19^,^20^. However, whether p38 MAPK signaling modulates MMP activity in ischemic stroke, which has a very different pathophysiology from cancer, and which types of MMPs are involved in this pathway in ischemic injury have rarely been reported.

Here, we report an ultrafast MMP activatable NIRF imaging probe with a relatively short activation time to achieve the real-time visualization of MMP activity in a mouse model of ischemic stroke. Then, we further test the potential utility of this probe in evaluating the effect of p38 MAPK inhibitor on the non-invasive regulation of MMP activity in the process of ischemic stroke.

## Results

### Characterization of the MMP-P12 probe

**Figure 1:**
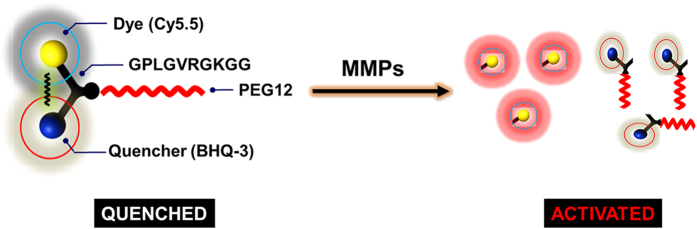
Chemical structure of the MMP-P12 probe. The side chain-protected MMP substrate GPL*GV*RGKGG was labeled with Cy5.5 as a near-infrared dye and was connected to BHQ-3 as a quencher. A specific size of PEG (PEG 12) was selected as a backbone of the quenched molecular beacon. The probe remained in a quenched state until it was degraded by activated MMPs under pathophysiological conditions and emitted intense fluorescent signals.

The chemical structure of the MMP activatable probe is shown in Fig. 1. The side chain-protected MMP substrate GPL*GV*RGKGG was labeled with Cy5.5 as a near-infrared dye and connected to BHQ-3 as a quencher. A specific size of PEG (PEG 12, molecular mass of 545 Da) was selected as a backbone of the quenched molecular beacon for its optimized stability, blood half-life and enzyme susceptibility, which was achieved by the molecular weight and steric hindrance. The probe remained in a quenched state until it was degraded by activated MMPs under pathophysiological conditions and emitted intense fluorescent signals.

### *In vivo* imaging and *ex vivo* bio-distribution of the MMP-P12 probe

**Figure 2:**
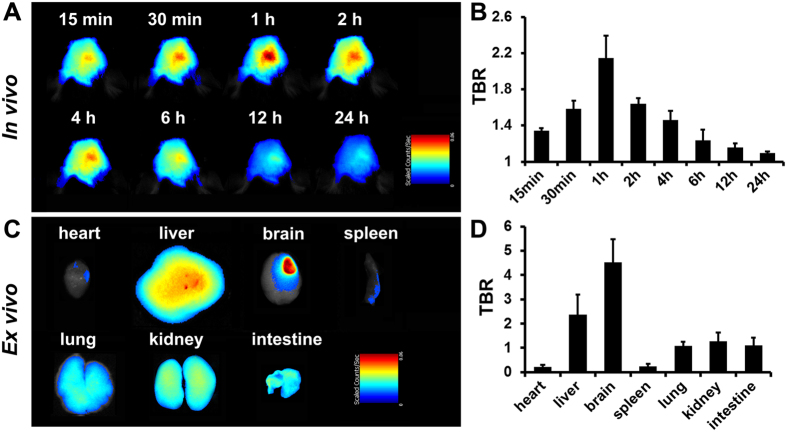
*In vivo* NIRF imaging and *ex vivo* bio-distribution of the MMP-P12 probe. (**A**) *In vivo* NIRF images of the ischemic brain from 15 min to 24 h after probe injection on day 7 post stroke and (**B**) quantitative target-to-background ratio (TBR) values (n = 3). (**C**) Bio-distribution of the probe in *ex vivo* organs 1 h after probe injection on day 7 post stroke and (**D**) quantitative TBR values of different organs in NIRF images (n = 3).

Serial *in vivo* NIRF images were acquired in the ischemic brain from 15 min to 24 h after probe injection (Fig. 2A) and showed significant signal enhancement in as little as 15 min, with peak signal intensity occurring at 1 h post-injection (Fig. 2B). *Ex vivo* NIRF imaging acquired 1 h after probe injection revealed the tissue distribution of the MMP-P12 probe, which primarily accumulated in the ischemic brain (Fig. 2C). The target-to-background ratio (TBR) quantified from the NIRF images was significantly higher in the brain than in other organs (Fig. 2D). These data demonstrate the efficiency of this probe in the real-time, non-invasive evaluation of MMP activity in the ischemic brain and offer an ultrashort activation time and a high target specificity.

### NIRF imaging of MMP activity was in accordance with stroke development

**Figure 3:**
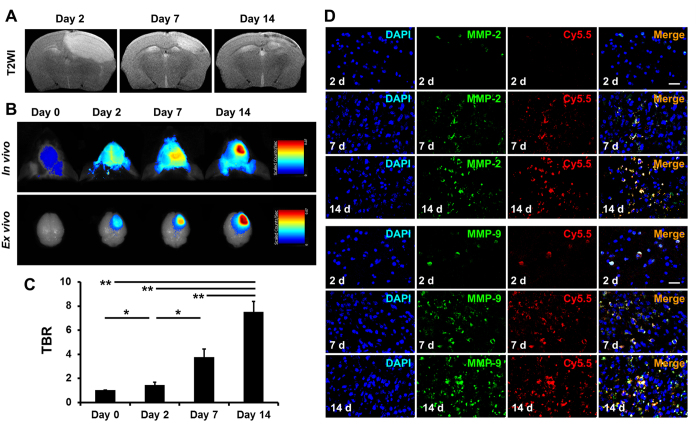
NIRF imaging of MMP activity was in accordance with stroke development. (**A**) Representative T_2_-weighted images after stroke. (**B**) *In vivo* and *ex vivo* NIRF images of the ischemic brain 1 h after probe injection and (**C**) quantitative target-to-background ratio (TBR) values in the ischemic brain 1 h after probe injection. (**D**) Immunofluorescence images of MMP-2 and MMP-9 indicate that probe activation is associated with the elevation in MMP activity during stroke development (n = 4–6 per group). Scale bar in D, 20 μm. *P < 0.05; **P < 0.01.

To dynamically evaluate MMP activity after stroke, magnetic resonance imaging (MRI), NIRF imaging and immunofluorescence imaging were performed at selected time points. The infarct areas were observed on T_2_-weighted imaging (Fig. 3A). *In vivo* and *ex vivo* NIRF images manifested gradually increased signal intensity in the ischemic brain from days 2 to 14 (Fig. 3B). The TBR values were significantly enhanced on days 7 and 14 compared with days 0 and 2 (Fig. 3C). Immunofluorescence imaging confirmed the increased expression of MMP-2 and MMP-9 in ischemic regions from days 2 to 14, and the activated MMP-2 and MMP-9 merged well with the Cy5.5 fluorescence of the MMP-P12 probe (Fig. 3D). Interestingly, the expression of MMP-9 was more pronounced than that of MMP-2. These data show that probe activation is tightly associated with MMP activity variations during stroke development and indicate that this probe is capable of dynamically visualizing MMP activity variations.

### Treatment with an MMP inhibitor reduced NIRF signals

**Figure 4:**
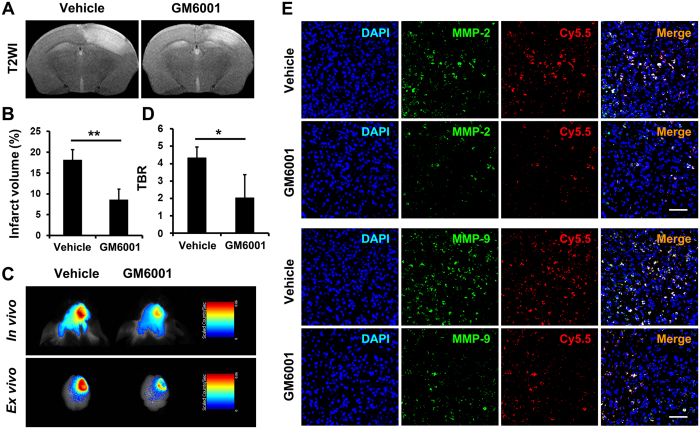
Treatment with an MMP inhibitor reduced the NIRF signals. (**A**) T_2_-weighted images and (**B**) related quantification of the infarct size of the ischemic brain on day 7 post stroke receiving vehicle or MMP inhibitor (GM6001) treatment once daily for 7 days. (**C**) *In vivo* and *ex vivo* NIRF images and (**D**) quantification of target-to-background ratio (TBR) values of the ischemic brain 1 h after MMP-P12 injection at the same time point. (**E**) Immunofluorescence images of MMP-2 and MMP-9 in the ischemic brain 1 h after probe injection confirmed the sensitivity of the MMP-P12 probe in quantifying reduced MMP activity in response to MMP-specific inhibitors on day 7 post stroke (n = 5 per group). Scale bar in E, 50 μm. *P < 0.05; **P < 0.01.

A broad-spectrum MMP inhibitor, GM6001, was administered to the mice once daily for 7 days after stroke. A significant reduction in the infarction size was observed after GM6001 treatment on T_2_-weighted MRI (Fig. 4A,B). *In vivo* and *ex vivo* NIRF images were acquired on day 7 (Fig. 4C), and the TBR values were significantly decreased after GM6001 treatment (Fig. 4D). Immunofluorescence imaging of the brain tissues showed that the amounts of MMP-2 and MMP-9 were significantly reduced after GM6001 treatment (Fig. 4E), thus confirming the sensitivity of this probe in quantifying the reduced MMP activity in response to MMP-specific inhibitors.

### Correlation between MMP expression and p38 MAPK activation

**Figure 5:**
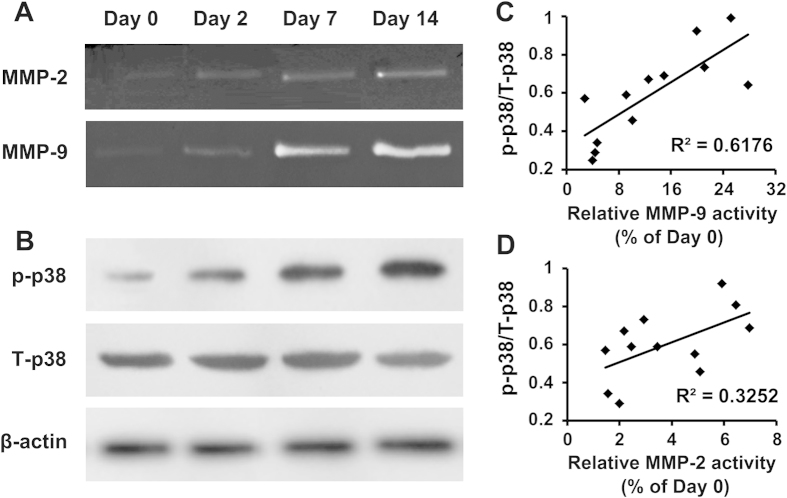
Correlation between MMP expression and p38 MAPK activation. (**A**) Gelatin zymography analysis of MMP-2 and MMP-9 protease activity and (**B**) western blotting of phosphorylated-p38 MAPK (p-p38) and total-p38 MAPK (T-p38) expression on days 0 (before stroke), 2, 7 and 14 after stroke. (**C**) The ratio of p-p38/T-p38 was significantly correlated with increased MMP-9 activity (R^2^ = 0.6176, *P* = 0.002; C), but showed no correlation with MMP-2 activity (R^2^ = 0.3252, *P* = 0.053; **D**) (n = 4 per group).

To identify the relationship between MMP activity and p38 MAPK activation in stroke mice, gelatin zymography was performed to detect the protease activity of MMP-2 and MMP-9, and western blotting was conducted to evaluate p38 MAPK expression. As shown in Fig. 5A, the MMP-9 activity was markedly increased from days 2 to 14 after stroke, whereas a significantly smaller increase in MMP-2 activity was observed compared with MMP-9 activity. Moreover, phosphorylated-p38 MAPK (p-p38) levels were also significantly increased from days 2 to 14 after stroke (Fig. 5B), and the ratio of p-p38 to total-p38 (p-p38/T-p38) was significantly correlated with increased MMP-9 expression (R^2^ = 0.6176, *P* = 0.002; Fig. 5C) but showed no significant correlation with MMP-2 activity (R^2^ = 0.3252, *P* = 0.053; Fig. 5D). These results suggested the involvement of the p38 MAPK pathway in MMP modulation after ischemic stroke, which was mainly achieved through the p38 MAPK-MMP-9 pathway.

### Inhibition of p38 MAPK improved stroke recovery

**Figure 6:**
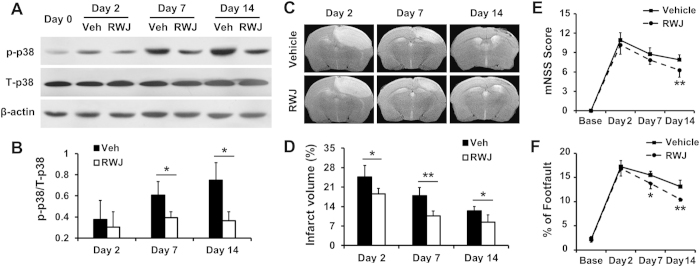
Inhibition of p38 MAPK improved stroke recovery. (**A**) Western blotting of phosphorylated-p38 MAPK (p-p38) and total-p38 MAPK (T-p38) and (**B**) quantitative analysis of the ratio of p-p38/T-p38 on days 0, 2, 7 and 14 with vehicle (Veh) or RWJ67657 (RWJ) administration. (**C**) T_2_-weighted images and (**D**) related quantification of infarct size in the ischemic brain after Veh or RWJ treatment. (**E**) mNSS score and (**F**) foot-fault analyses in stroke mice (n = 6 per group). *P < 0.05; **P < 0.01.

To study the effects of the p38 MAPK inhibitor on ischemic stroke, RWJ67657 was administered daily to stroke mice; a group of vehicle-treated mice was used as the control. RWJ67657 treatment significantly decreased the protein expression of p-p38 MAPK in the ischemic brain compared with the vehicle group on days 7 and 14 (Fig. 6A,B). *In vivo* T_2_-weighted MRI showed a significant reduction of infarct volume after RWJ67657 treatment compared with the vehicle group from day 2 to day 14 (Fig. 6C,D). In behavior tests, a significant reduction of the neurological severity score (mNSS) was observed on day 14 after RWJ67657 treatment compared with the vehicle group (Fig. 6E), and the percentage of foot-fault was significantly lower in the mice treated with RWJ67657 on day 7 and 14 compared with the vehicle group (Fig. 6F), which indicated that the p38 MAPK inhibitor could promote functional recovery after ischemic stroke.

### Inhibition of p38 MAPK reduced MMP activity

**Figure 7:**
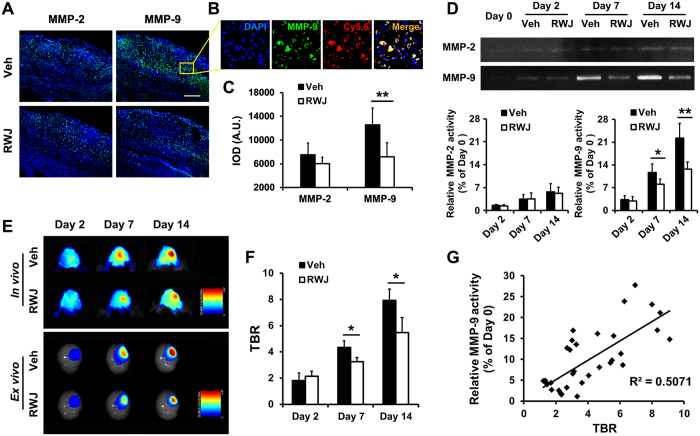
Inhibition of p38 MAPK reduced MMP activity and image-guided MMP activity tracking in response to p38 MAPK inhibition. (**A**) Immunofluorescence imaging of MMP-2 and MMP-9 in the ischemic brain on day 14 after ischemic stroke with vehicle (Veh) or RWJ67657 (RWJ) administration for 14 days. (**B**) The immunofluorescence staining of MMP (green) merged well with Cy5.5 (red) in the MMP-P12 probe, indicating that the NIRF imaging of this probe was capable of reflecting the activated MMPs in the ischemic brain. (**C**) Quantitative integral optical density (IOD) values in the ischemic brain. (**D**) Gelatin zymography and quantitative analysis of MMP-2 and MMP-9 activity showed that only MMP-9 was significantly decreased after daily RWJ treatment on days 7 and 14. No statistically significant difference was detected for MMP-2 activity after RWJ treatment at any time point. (**E**) *In vivo* and *ex vivo* NIRF images and (**F**) target-to-background ratio (TBR) quantification of the ischemic brain showed significant TBR reductions in the RWJ group compared with the Veh group on days 7 and 14 after daily treatment. (**G**) The TBR values in NIRF imaging were significantly correlated with the increased MMP-9 activity (R^2^ = 0.5071, *P* < 0.001), thus demonstrating the high sensitivity of this probe in the non-invasive monitoring of MMP variations in response to therapeutic interventions (n = 6 per group). Scale bar in C, 200 μm. *P < 0.05; **P < 0.01.

To study the efficacy of the p38 MAPK inhibitor on MMP activity modulation in ischemic stroke, the immunofluorescence staining of MMP-2 and MMP-9 expression was obtained on day 14 post-stroke (Fig. 7A). Interestingly, only MMP-9 staining showed a significantly decreased integral optical density (IOD) value in RWJ67657-treated mice compared with the vehicle-treated group; although there was still a slight reduction in the IOD value after RWJ67657 treatment in MMP-2 staining, no statistical significance was observed (Fig. 7C). Meanwhile, the expression of MMP (green) merged well with the Cy5.5 (red) in the MMP-P12 probe (Fig. 7B), thus demonstrating that the NIRF imaging of this probe was able to reflect the activated MMPs in the ischemic brain. Moreover, the MMP-9 activity was significantly decreased in the gelatin zymography analysis after RWJ67657 treatment compared with the vehicle group on days 7 and 14 (Fig. 7D), while no statistically significant difference was detected in MMP-2 activity at any time point. These data suggest that the p38 MAPK inhibitor inhibits MMP activity in ischemic stroke mainly through the modulation of MMP-9, which in this work has uncovered a novel MMP-associated regulatory pathway of ischemic stroke and brings new opportunities for stroke therapy.

### Image-guided MMP activity tracking in response to p38 MAPK inhibition

To evaluate the efficacy of the MMP-P12 probe in monitoring the changes in MMP activity after RWJ67657 administration, *in vivo* and *ex vivo* NIRF images were acquired on days 2, 7 and 14 (Fig. 7E). Notably, significant TBR reductions were observed in the RWJ67657 group compared with the vehicle group on days 7 and 14 (Fig. 7F), and the TBR values in NIRF imaging were significantly correlated with the increased MMP-9 activity as detected in gelatin zymography (R^2^ = 0.5071, *P* < 0.001; Fig. 7G). All of these results suggested that this novel ultrafast MMP activatable probe was sufficient to non-invasively measure MMP activity alterations after ischemic stroke, which provided an ultrasensitive optical imaging strategy for monitoring MMP variations in response to MMP-mediated therapeutic interventions.

## Discussion

Herein, we report an ultrafast and sensitive MMP-activatable NIRF imaging probe for monitoring MMP activity *in vivo* in a mouse model of ischemic stroke. Using this probe in our work, we accomplished the real-time visualization of MMP activity after stroke and demonstrated that the inhibition of the p38 MAPK signal transduction pathway was capable of down-regulating MMP activity after cerebral ischemia.

Ischemic stroke is among the leading causes of death and disability worldwide. Although a number of clinical trials of stroke treatment have been conducted, the only FDA-approved treatment for improving stroke outcomes is recombinant tissue plasminogen activator (rtPA)^2^. Unfortunately, the proportion of patients eligible for thrombolysis is very small due to the narrow time window and harsh exclusion criteria of rtPA. Thus, alternative types of therapeutic management are needed for stroke treatment.

In ischemic stroke, MMPs control extracellular matrix degradation and neurovascular remodeling, and their activity acutely disrupts BBB and promotes brain edema and hemorrhaging^21^,^22^. Among all MMP subtypes, MMP-2 and MMP-9 are the most studied MMPs in cerebrovascular disease. The accumulated data suggest that MMP-9 is markedly increased after stroke and is associated with acute BBB rupture and resultant neuron damage^23^. Mice deficient in MMP-9 have a smaller cerebral infarct size compared with control mice^24^, and the inhibition of MMP-9 activity with MMP inhibitors significantly prevents BBB breakdown^25^. Although several studies proposed that early upregulation of MMP-9 was related to severe ischemic stroke outcomes^26^,^27^, other studies also discovered a delayed enhancement of MMP-9 activity in the periphery of cortical infarction by days 7–14, which was instead associated with neurovascular remodeling and stroke recovery^28^,^29^. Lucivero *et al.* proposed that plasma MMP-9 level was significantly increased in the later phase at 7 d after stroke^30^, and Castellanos *et al.* showed that the acute increase of MMP-9 activity was significantly correlated with haemorrhagic transformation in stroke patients^31^. These studies suggest that the exact temporal and spatial distribution regarding MMP-9 activation after stroke remains unclear, which may be associated with multi-factors such as the severity of stroke and complications thereafter. Compared with MMP-9, the elevation of MMP-2 activity is previously considered much slower and has lower potential for contributing to acute brain damage^32^. Several studies proposed that MMP-2 may have a positive effect in the repairing phases of ischemia^23^. However, other researchers supported that the initial opening of BBB was correlated with elevated levels MMP-2 at 3 h after ischemia reperfusion, which indicated a deleterious role of MMP-2 in the early stage of stroke^33^. Due to these controversial results of MMPs activation after cerebral ischemia, and considering that MMPs represent ideal therapeutic targets for ischemic stroke, efficient method to non-invasively visualize MMP activity is of great importance. However, the currently available methods for the detection of MMP activity and expression, such as gelatin zymography, PCR and western blotting, are all invasive. The lack of a non-invasive imaging strategy largely limits deeper research on MMPs in both preclinical and clinical studies.

Recently, the emerging molecular imaging techniques have brought new opportunities for the non-invasive evaluation of histopathological alterations in various diseases. Imaging modalities such as positron emission tomography^34^, MRI^35^, and NIRF imaging^36^ have already been used in animal studies and clinical practice. Of all the available imaging modalities, NIRF imaging possesses high sensitivity, low cost and nonionizing radiation, which is desirable for *in vivo* applications. Indeed, NIRF imaging has already been applied for the visualization of MMP activity in various diseases, including ischemic stroke^14^,^37^,^38^,^39^. For example, the commercially available MMPSense 680™ (Perkin Elmer Inc., Boston, MA, USA) has been used to evaluate MMP expression in a mouse model of stroke^14^. However, it requires a long activation time (24 h) for *in vivo* imaging due to its high-molecular-weight polymer backbone, which is an inherent defect with respect to real-time evaluations. Philip *et al.* reported an optimized MMPSense 750 with a shorter activation time (3 h) for the detection of MMPs in cerebral ischemia; however, it offered a low signal intensity and can only be detected after skull removal^16^. To optimize MMP activated probe, we synthesized a sequence of MMP activated probes conjugated with different sizes of PEG in our previous studies, and we observed that different PEG lengths appreciably affected the half-life and enzyme degradation efficiency of the probe^40^,^41^. By comprising those probes with different PEG backbones and MMPSense 680™, MMP-P12 with PEG12 was established for its best sensitivity, highest signal contrast and fastest enzyme recognition time. Moreover, the probe was further optimized by replacing _L_-lysine with _D_-lysine in the MMP substrate sequence, thereby diminishing nonspecific cleavage by other proteases and achieving enhanced target-to-background contrast^41^.

Regardless of these advantages, MMP-P12 has not yet been tested in ischemic stroke. Considering the critical roles of MMPs in stroke progression, non-invasive detection of MMPs is extremely important. Thus, we further tested MMP-P12 in a mouse model of photothrombotic stroke in the current study. Notably, the finalized MMP-P12 probe was sufficient for visualizing activated MMPs as early as 15 min post probe injection, offering the highest signal intensity at 1 h; such results make this probe much faster-acting than the current existing MMP activatable NIRF probes. In addition, the signal intensity was far stronger in the ischemic brain than in other organs, such as the liver and spleen, and that the use of a specific MMP inhibitor GM6001 significantly inhibited the activation of MMPs and lowered the signal contrast, which indicated the unique specificity of this probe to be cleaved by local MMP enzymes in the ischemic brain. Control study was also conducted by injecting MMP-P12 probe in a group of naïve mice (Supplementary Figure S1), whereas no significant fluorescent signal was observed in the naïve group compared with the stroke group in all organs including brain and liver, which further demonstrated the stability of this probe to avoid nonspecific degradation. Moreover, the high sensitivity and target-to-background contrast of MMP-P12 in quantifying the alterations of activated MMPs in ischemic regions highlight its usefulness in achieving non-invasive evaluation of MMP activity and drug screening in ischemic stroke. Owing to the emerging basic and clinical studies of MMP regulation, the establishment of this probe will contribute to a better understanding of the function of MMPs in cerebral ischemia as well as the generation of new therapeutic strategies.

MMP inhibitors have long been applied to stroke treatment and are considered effective in a wide range of animal studies where they prevent neuron necrosis, reduce infarction size and alleviate neurological dysfunction^4^,^5^. Studies have reported that the early application of an MMP inhibitor can reduce rtPA-mediated mortality after cerebral ischemia through BBB closure^42^. The endogenous MMP inhibitors, known as the tissue inhibitor of MMPs (TIMPs), are also capable of protecting against BBB disruption and play a neuroprotective role in cerebral ischemia^43^. Due to the positive results observed for MMP inhibitors in pre-clinical experiments, over 50 MMP inhibitors have been evaluated in clinical trials^7^. However, to date, all of these trials have failed due to side effects and dose-limited ineffectiveness. In a phase I clinical trial of non-small cell lung cancer, significant musculoskeletal toxicity was observed when the combination of an MMP inhibitor and conventional anti-cancer drugs was used^44^. In a phase III trial of breast cancer, an MMP inhibitor was ineffective at doses already associated with severe side effects^45^. The exploration of optimized MMP inhibitors and novel regulatory agents for targeting MMPs are urgently needed to overcome these obstacles.

P38 MAPK signaling is involved in various cellular processes, including development, proliferation, differentiation and apoptosis. Inhibitors targeting the p38 MAPK pathway have been proven effective in inhibiting inflammation^46^ and caspase-3 activation^47^. In ischemic stroke, the activation of p38 MAPK was observed early in the postischemic brain and maintained high expression in the long-term, which was associated with poor recovery^48^ and implied the therapeutic potential of p38 MAPK inhibition after infarction with a wide time window^49^. Recently, activated p38 MAPK was observed to be associated with the regulation of MMP activity in tumors, and p38 MAPK signaling was shown to modulate cancer invasion partly through an MMP-associated mechanism^17^,^18^. However, the involvement of the p38 MAPK pathway in MMP activation after cerebral ischemia remains unclear, and which MMP subtype is involved in the neuroprotective mechanism of p38 MAPK inhibitors in the ischemic brain is also not yet clear.

In this study, a significant linear correlation was observed between the activated p38 MAPK protein expression and MMP-9 activity, which was much higher than the correlation between p38 MAPK and MMP-2 activity. Then, the p38 MAPK inhibitor RWJ67657 was applied in ischemic stroke mice to determine its effect on the regulation of MMP activity. Interestingly, RWJ67657 significantly inhibited MMP-9 activity on days 7 and 14 post-stroke. Although a slight reduction in MMP-2 expression was also observed after RWJ67657 treatment, this difference did not reach statistical significance. These results revealed the involvement of the p38 MAPK-MMP-9 pathway in the progression of ischemic stroke, representing a novel therapeutic target for stroke treatment. More importantly, the MMP-P12 probe efficiently measured the changes in MMP activity after RWJ67657 administration with high sensitivity and accuracy, thus providing a powerful real-time imaging strategy for monitoring the dynamic evolution of MMP activity as well as drug screening in preclinical settings.

In summary, the present study provides a novel fast and ultrasensitive NIRF imaging probe for the non-invasive and dynamic visualization of MMP activity after ischemic stroke. Using this probe, we successfully demonstrated that the regulation of the p38 MAPK pathway was capable of modulating MMP activity after ischemic stroke, revealing a novel regulatory mechanism of postischemic brain damage and overcoming the limitations of traditional therapeutic strategies of MMP inhibitors using an *in vivo* molecular imaging method. Considering the increasing number of studies on MMP-mediated mechanisms and MMP inhibitors in basic research and clinical practice, the establishment of this novel NIRF imaging strategy will contribute to a better understanding of the complex roles of MMPs in cerebral ischemia and will accelerate the development of optimized MMP-specific inhibitors, as well as facilitate further explorations of novel MMP-associated pathways and alternative therapeutic interventions.

## Methods

A detailed methodology is provided in the Supplementary information files.

### Ethics statement

All experimental protocols were approved by the Institutional Animal Use and Care Committee (IACUC) of the Medical School of Southeast University (Nanjing, Jiangsu, China; approval ID: SYXK-20130413275). The surgeries and procedures were performed in strict accordance with the Guidelines for the National Care and Use of Laboratory Animals by the National Animal Research Authority (China).

### Photothrombotic stroke model

A total of 80 male C57BL/6 mice (8-week-old; Academy of Military Medical Science, Beijing, China) were purchased and kept on a 12/12 h light-dark cycle with food and water freely available during the experimental period. Anesthesia was induced with 2% isoflurane (KeYuan, Shandong, China) and maintained with 1% isoflurane using a gas anesthesia mask. Cerebral ischemic stroke was induced in mice via photothrombosis, as previously described by our group and others^50^,^51^,^52^. T_2_-weighted MRI was conducted to verify photothrombotic ischemia at 1 day post-surgery.

### Synthesis of the MMP activatable probe

The MMP activatable probe (MMP-P12) was synthesized by the conjugation of an NIRF dye Cy5.5 (GE Healthcare, Piscataway, NJ, USA), an MMP substrate GPL*GV*RGKGG, a black hole quencher-3 (BHQ-3; Bioresearch Technologies, Navato, CA, USA) and amino PEG analogs (Quanta Biodesign, Powell, OH, USA), as described in our previous studies^40^,^41^. Briefly, side chain-protected MMP substrate was first synthesized by standard solid-phase Fmoc peptide chemistry using an acid-sensitive resin. After Cy5.5 labeling, amino PEG with a molecular weight of 545 g/mol was conjugated to the C-terminus of the peptide. Finally, side chain protection groups were removed with a trifluoroacetic acid (TFA) cocktail, allowing for BHQ-3 conjugation. The final product was purified by high-performance liquid chromatography (HPLC) and confirmed by liquid chromatography-mass spectrometry (LC-MS).

### Animal experimental protocol

The animal experiments mainly consisted of four parts. First, to evaluate the probe’s *in vivo* behavior, serial NIRF imaging was performed from 15 min to 24 h after probe injection (15 min, 30 min, 1 h, 2 h, 4 h, 8 h, 12 h and 24 h) on 7 days post-stroke (n = 3). The time point with peak signal enhancement was selected for further imaging. Second, to non-invasively assess the efficiency of MMP-Cy5.5 in evaluating the dynamic MMP activity variations during stroke progression, photothrombotic stroke mice were separately injected with the probes on days 0 (before stroke), 2, 7 and 14 after stroke, after which NIRF imaging (n = 4–6 per group) was performed. Third, to evaluate the probe’s sensitivity in response to an MMP-specific inhibitor, a broad-spectrum MMP inhibitor, GM6001 (Selleck Chemicals, Huston, Texas, USA), was intraperitoneally administered immediately after stroke and once daily for 7 days (50 mg/kg/day). The GM6001-negative control group received the same dose of N-t-butoxycarbonyl-L-leucyl-L-tryptophan methylamide (vehicle; Selleck Chemicals). NIRF images were performed on day 7 after treatment (n = 5 per group). Finally, for the therapeutic analysis of the p38 MAPK inhibitor on stroke recovery and MMP regulation, RWJ67657 (50 mg/kg/d; Santa Cruz, Texas, USA) was administered daily to stroke mice by gavage immediately after stroke. A group of stroke mice received the same dose of 5% methylcellulose (vehicle, placebo group). NIRF images were acquired on days 0, 2, 7 and 14 (n = 6 per group) to test the feasibility of this probe in image-guided therapy. In each experimental group, *in vivo* MRI was simultaneously acquired to calculate the infarct volume, and laser-scanning confocal microscopy (Olympus, Tokyo, Japan) was performed on *ex vivo* brain tissues after immunofluorescence staining. Gelatin zymography and western blotting were conducted separately to evaluate MMP activity and p38 MAPK expression.

### NIRF imaging

*In vivo* and *ex vivo* NIRF imaging was performed using a Maestro *In-Vivo* Imaging System (CRi, Woburn, MA, USA). The MMP-Cy5.5 probe (8 nM, 200 μL in PBS, pH 7.4) was injected *via* the tail vein. Mice were anesthetized with 2% isoflurane (KeYuan) and placed in the prone position on the examination table. The fluorescence of the MMP-Cy5.5 probe was detected by two filter sets (Cy5.5: excitation = 675 nm; emission = 695 nm long pass). After *in vivo* NIRF imaging, all mice were euthanized, and the brain, heart, spleen, liver, kidneys and lungs were surgically dissected for *ex vivo* NIRF imaging using the same parameters. The quantification of the NIRF signal intensity was achieved from *ex vivo* tissues, as mentioned in our previous study^51^,^52^.

### MRI measurements

*In vivo* MRI was performed using a 7.0-Tesla small animal MR scanner (Bruker PharmaScan, Germany), as previously described^51^,^52^. T_2_-weighted images were acquired on days 2, 7 and 14 post-ischemia to calculate the percentage of infarct volume.

### Behavioral tests

The modified neurological severity score (mNSS) and foot-fault test were assessed before ischemia (base) and on days 2, 7 and 14 after ischemia by two investigators who were blinded to the experimental groups, in approach that we previously reported^51^.

### Immunofluorescence staining

Brain tissues were dissected and fixed in 4% paraformaldehyde for 24 h. After dehydration, cryostat-sectioned slices (4 μm) were incubated with rabbit anti-mouse antibodies against MMP-2 and MMP-9 (Abcam, Hong Kong, China). Then, the slides were stained with a goat anti-rabbit Alexa Fluor 488 polyclonal antibody (Life Technologies, Grand Island, NY, USA). Nuclei were counterstained with DAPI (Sigma-Aldrich, St. Louis, MO, USA). For quantitative analysis, the slices were scanned by laser-scanning confocal microscopy, and observers were blinded to the experimental groups.

### Gelatin zymography

Immediately after the mice were sacrificed, the ischemic brain hemispheres were harvested and frozen. To analyze the protease activity of MMP-2 and MMP-9, gelatin zymography was performed on days 0 (before stroke), 2, 7 and 14 after stroke with or without RWJ67657 administration, as described^18^.

### Western blotting

The protein concentrations of each sample were calculated using the bicinchoninic acid method. The protein was separated by SDS-PAGE and transferred to a polyvinylidenefluoride membrane (Millipore, Boston, Massachusetts, USA). After blocking with 5% skim milk (BD Bioscience, San Diego, USA), the samples were incubated with primary rabbit anti-mouse antibodies to T-p38 (total p38) MAPK, p-p38 (phosphorylated-p38) MAPK, and β-actin (Abcam) at 4°C overnight followed by incubation with horseradish peroxidase-conjugated secondary antibody (Life Technologies). The protein levels (band densities) in each sample were quantified using Image J software (NIH, Bethesda, MD, USA).

### Data analysis

Data are expressed as the mean ± standard deviation (SD). For statistical analysis, one-way analysis of variance (ANOVA) was applied for multi-group comparisons. *Post hoc* analysis with appropriate Bonferroni correction was conducted, and two-sided testing was used. The Student’s t-test was used for two-group comparisons. The correlation analysis was performed using Pearson’s correlation coefficients. All statistical tests were performed using SPSS software (version 20), and values of *P* < 0.05 were considered statistically significant.

## Additional Information

**How to cite this article**: Chang, D. *et al.* Role of P38 MAPK on MMP Activity in Photothrombotic Stroke Mice as Measured using an Ultrafast MMP Activatable Probe. *Sci. Rep.* **5**, 16951; doi: 10.1038/srep16951 (2015).

## Supplementary Material

Supplementary Information
