# Supplementary material for: Role of P38 MAPK on MMP Activity in Photothrombotic Stroke Mice as Measured using an Ultrafast MMP Activatable Probe
Source: Sci Rep. 2015 Nov 19;5:16951. doi: 10.1038/srep16951 (PMC4652271; doi:10.1038/srep16951)
Supplement: Supplementary Information [file srep16951-s1.doc]

**ONLINE DATA SUPPLEMENT**

**Role of P38 MAPK on MMP Activity in Photothrombotic Stroke Mice as Measured using an Ultrafast MMP Activatable Probe**

*Di Chang, MD^1^; Yuan-Cheng Wang, MD^1^; Ying-Ying Bai, MD^1^;* *Chun-Qiang Lu, MD**^1^;* *Ting-Ting Xu, MD^1^; Lei Zhu, PhD^2^*; Shenghong Ju, MD, PhD^1^**

^1^ Jiangsu Key Laboratory of Molecular and Functional Imaging, Department of Radiology, Zhongda Hospital, Medical School of Southeast University, Nanjing 210009, China;

^2^ State Key Laboratory of Molecular Vaccinology and Molecular Diagnostics & Center for Molecular Imaging and Translational Medicine, School of Public Health, Xiamen University, Xiamen, Fujian, 361005, China.

***Address for Correspondence:**

Shenghong Ju, MD, PhD, Jiangsu Key Laboratory of Molecular and Functional Imaging, Department of Radiology, Zhongda Hospital, Medical School of Southeast University, 87 Ding Jia Qiao Road, Nanjing 210009, China. Tel: +86 25 83272115; Fax: +86 25 83311083; E-mail: [jsh0836@hotmail.com](mailto:jsh0836@hotmail.com)

Lei Zhu, PhD, State Key Laboratory of Molecular Vaccinology and Molecular Diagnostics & Center for Molecular Imaging and Translational Medicine, School of Public Health, Xiamen University, Xiamen, Fujian, 361005, China. E-mail: [lei.zhu@xmu.edu.cn](mailto:lei.zhu@xmu.edu.cn)

**Supplementary Methods**

**MRI parameters**

*In vivo* MRI was performed on a 7.0-Tesla small animal MR scanner (Bruker PharmaScan, Germany). The mice were anesthetized with 2% isoflurane delivered through a nose cone, and their respiratory rate and body temperature were monitored via a physiology monitor. T2-weighted imaging was conducted at days 1, 2, 7 and 14 using a two-dimensional fast-spin echo sequence (2,000/50 msec of repetition time/echo time, 1 average). Twelve axial slices with a slice thickness of 1 mm, matrix of 256 × 256 and a field of view of 20 × 20 mm were positioned over the brain, excluding the olfactory bulb. The total scan time was 1 min 13 s.

The percentage of infarct volume was calculated on T2-weighted imaging. Briefly, the percentage of infarct volume was presented as the lesion volume/the contralateral hemisphere volume covering the whole slices of T2-weighted images. The lesion volume was acquired based on the high signal area of T2-weighted images from which the artifact of brain edema was subtracted, i.e., the non-lesioned ipsilateral hemisphere was subtracted from the contralateral hemisphere. Infarct volume was traced and calculated using the Image J software (National Institutes of Health, USA).

**Photothrombotic stroke model**

Cerebral ischemic stroke was induced *via* photothrombosis. Briefly, mice were anesthetized with 2% isoflurane and maintained with 1% isoflurane using a gas anesthesia mask. Rose Bengal solution (100 mg/kg, 10 mg/mL in saline, Sigma-Aldrich, St. Louis, MO, USA) was administered intraperitoneally 5 min before illumination. For illumination, a cold light source (KL1500 LCD, Zeiss, Germany) with a 4-mm-diameter fiber was positioned 2 mm to the right of the bregma after the hair was shaved. The brain was illuminated for 15 min to allow the injected Rose Bengal solution to induce singlet oxygen, which resulted in the activation of platelets with consequent damages and occluding vascular endothelium, which mimicked the traditional ischemic stroke model. T2-weighted MRI was conducted to verify photothrombotic ischemia at 24 h post-surgery.

**Quantification of NIRF signal**

*In vivo* and *ex vivo* NIRF images were performed using a Maestro *In-Vivo* imaging system. To quantify the NIRF signal intensity, the fluorescence signal from each optical image was extracted from the autofluorescence signal based on spectral patterns using the multi-spectral imaging capabilities implemented in the Maestro 2.10.0 software. Then, the NIRF signal intensity from the explanted organs was quantified as counts per second per pixel using CRi Maestro software. Rectangular regions of interests (ROIs) were manually drawn on the stroke regions of the right hemisphere and then copied to the corresponding regions of the contralateral hemisphere on *ex vivo* images. The target-to-background ratio (TBR) was calculated as follows: (ROI value from the right hemisphere) / (ROI value from the left hemisphere).

**Behavioral tests**

Behavioral tests were performed on days 2, 7 and 14 after stroke. Before the induction of ischemia, all mice received training on the grid for balance test. Animals not achieving the criteria were excluded. The modified neurological severity score (mNSS) and foot-fault test were performed before ischemia (base) and on day 2, 7 and 14 after ischemia by two investigators who were blinded to the experimental groups. Briefly, the mNSS test consists of motor, sensory, reflex and balance tests, and the neurological function is graded on a scale of 0 to 18 (normal score, 0; maximal deficit score, 18). In assessing the severity scores of injury, 1 score point is awarded for inability to perform the test or a lack of test reflexes; thus, the higher the score, the more severe that the injury is. For the foot-fault test, each mouse was videotaped while walking on the grid freely for 5 min. With each weight-bearing step, a paw that fell between the wire grid lines was recorded as a foot fault. The percentage of foot-faults of the left paw to total steps was determined.

**Gelatin zymography**

The brain hemispheres with ischemia were dissected and homogenized in a lysis buffer. After electrophoresis, gels were incubated with 1 × renaturation buffer for 30 min, followed by overnight incubation in a developing buffer at 37 °C. Then, the bands were stained with a resolution of Coomassie blue R-250 for 1 h and destained with distilled water for another 2 h. The presence of enzyme activity was conﬁrmed by the appearance of white bands on a blue background. For visual clarity within ﬁgures, the images were converted to gray-scale and inverted (Adobe Photoshop), so digested bands appear as black on a white background. Semi-quantitative densitometry was performed using Image J 1.4 software (NIH, USA), and the results were normalized to the values of day 0 as a control.

**Supplementary Figures**


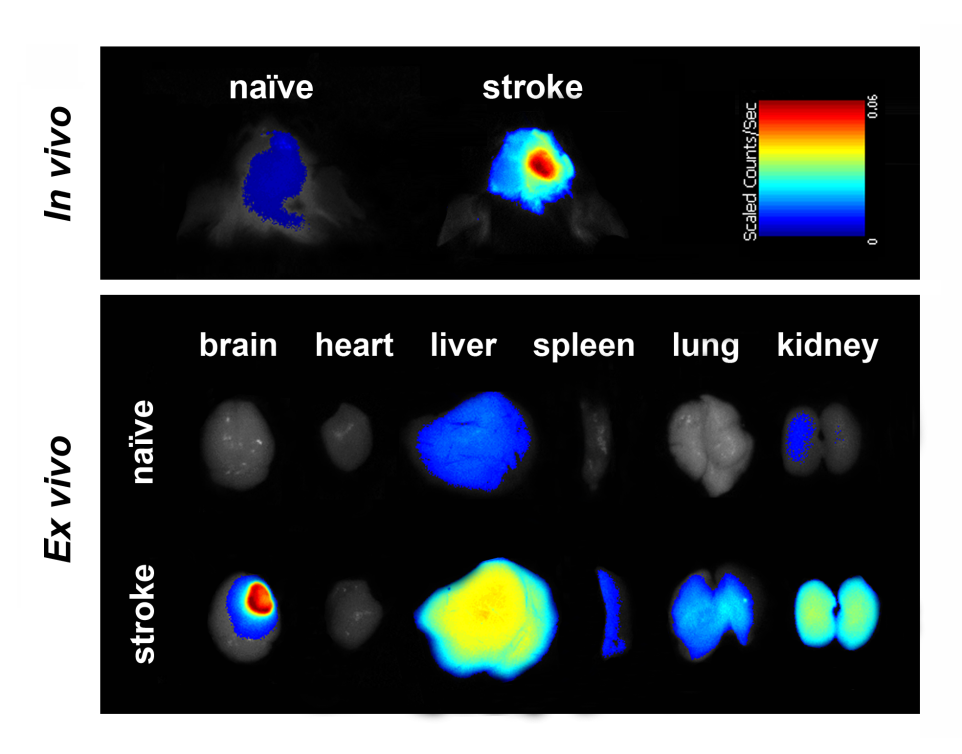


Supplementary Figure S1. The biodistributions of the MMP-P12 probe 1 h post injection separately in naïve mice or photothrombotic stroke mice at day 7 after stroke. No significant fluorescent signal was observed in the brain and also other organs in the naïve mice compared with the stroke mice, which demonstrated that the basal level of MMPs activity is low in the naïve mice and the probe can be specifically degraded by the activated MMPs in injured tissues.


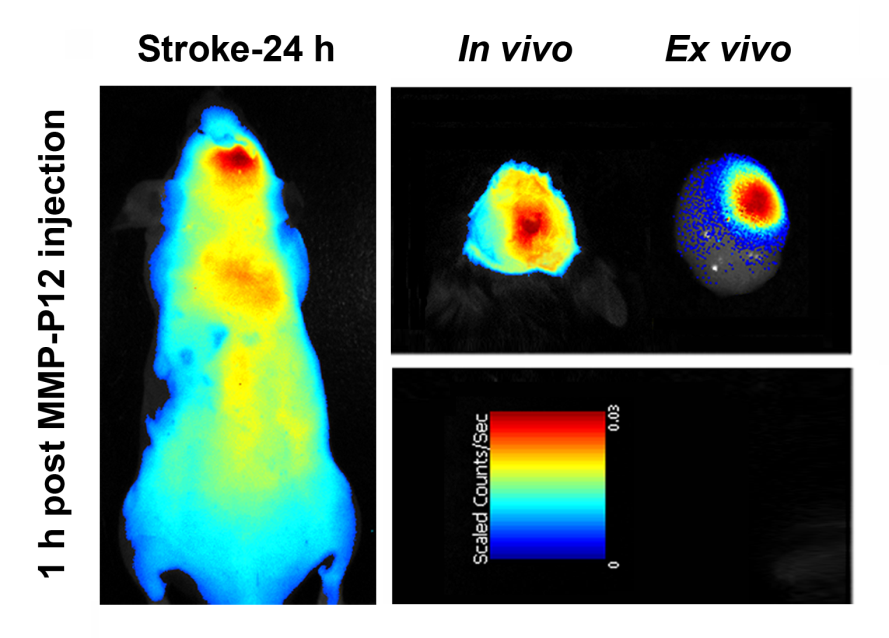


Supplementary Figure S2. MMP activity at 24 h post photothrombotic stroke. MMP activity at 24 h post photothrombotic stroke was detected by *in vivo* and *ex vivo* NIRF imaging 1 h after the MMP-P12 probe injection. Significant fluorescence signal contrast was observed at 24 h after photothrombotic stroke, which demonstrated that this probe was sufficient to detect MMP activity during acute time period after stroke.
